# Effect of Blueberry Supplementation on a Diet-Induced Rat Model of Prediabetes—Focus on Hepatic Lipid Deposition, Endoplasmic Stress Response and Autophagy

**DOI:** 10.3390/nu16040513

**Published:** 2024-02-13

**Authors:** Gonçalo Ferreira, Pedro Vieira, André Alves, Sara Nunes, Inês Preguiça, Tânia Martins-Marques, Tânia Ribeiro, Henrique Girão, Artur Figueirinha, Lígia Salgueiro, Manuela Pintado, Pedro Gomes, Sofia Viana, Flávio Reis

**Affiliations:** 1 Institute of Pharmacology & Experimental Therapeutics & Coimbra Institute for Clinical and Biomedical Research (iCBR), Faculty of Medicine, University of Coimbra, 3000-548 Coimbra, Portugal; goncalocrferreira@gmail.com (G.F.); pedro.vieira@uc.pt (P.V.); alves.andrefb@gmail.com (A.A.); sara.nunes20@gmail.com (S.N.); i.preguica@campus.fct.unl.pt (I.P.); taniamarques241@gmail.com (T.M.-M.); hmgirao@fmed.uc.pt (H.G.); pgomes70@gmail.com (P.G.); sofia.viana@uc.pt (S.V.); 2 CIBB—Center for Innovative Biomedicine and Biotechnology, University of Coimbra, 3004–504 Coimbra, Portugal; 3 Clinical Academic Center of Coimbra (CACC), 3004-531 Coimbra, Portugal; 4 Polytechnic Institute of Coimbra, ESTESC-Coimbra Health School, Pharmacy, 3045-043 Coimbra, Portugal; 5 CBQF—Centro de Biotecnologia e Química Fina—Laboratório Associado, Escola Superior de Biotecnologia, Universidade Católica Portuguesa, Rua Diogo Botelho 1327, 4169-005 Porto, Portugal; tribeiro@ucp.pt (T.R.); mpintado@ucp.pt (M.P.); 6 Faculty of Pharmacy, University of Coimbra, 3000-548 Coimbra, Portugal; amfigueirinha@ff.uc.pt (A.F.); ligia@ff.uc.pt (L.S.); 7 LAQV, REQUIMTE, Faculty of Pharmacy, University of Coimbra, 3000-548 Coimbra, Portugal; 8 CERES, Chemical Engineering and Renewable Resources for Sustainability, Department of Chemical Engineering, University of Coimbra, 3030-790 Coimbra, Portugal; 9 Department of Biomedicine, Faculty of Medicine, University of Porto, 4200-319 Porto, Portugal

**Keywords:** blueberry supplementation, prediabetes, diet-induced rat model, hepatic endoplasmic stress response and autophagy

## Abstract

Blueberries, red fruits enriched in polyphenols and fibers, are envisaged as a promising nutraceutical intervention in a plethora of metabolic diseases. Prediabetes, an intermediate state between normal glucose tolerance and type 2 diabetes, fuels the development of complications, including hepatic steatosis. In previous work, we have demonstrated that blueberry juice (BJ) supplementation benefits glycemic control and lipid profile, which was accompanied by an amelioration of hepatic mitochondrial bioenergetics. The purpose of this study is to clarify the impact of long-term BJ nutraceutical intervention on cellular mechanisms that govern hepatic lipid homeostasis, namely autophagy and endoplasmic reticulum (ER) stress, in a rat model of prediabetes. Two groups of male Wistar rats, 8-weeks old, were fed a prediabetes-inducing high-fat diet (HFD) and one group was fed a control diet (CD). From the timepoint where the prediabetic phenotype was achieved (week 16) until the end of the study (week 24), one of the HFD-fed groups was daily orally supplemented with 25 g/kg body weight (BW) of BJ (HFD + BJ). BW, caloric intake, glucose tolerance and insulin sensitivity were monitored throughout the study. The serum and hepatic lipid contents were quantified. Liver and interscapular brown and epidydimal white adipose tissue depots (iBAT and eWAT) were collected for histological analysis and to assess thermogenesis, ER stress and autophagy markers. The gut microbiota composition and the short-chain fatty acids (SCFAs) content were determined in colon fecal samples. BJ supplementation positively impacted glycemic control but was unable to prevent obesity and adiposity. BJ-treated animals presented a reduction in fecal SCFAs, increased markers of arrested iBAT thermogenesis and energy expenditure, together with an aggravation of HFD-induced lipotoxicity and hepatic steatosis, which were accompanied by the inhibition of autophagy and ER stress responses in the liver. In conclusion, despite the improvement of glucose tolerance, BJ supplementation promoted a major impact on lipid management mechanisms at liver and AT levels in prediabetic animals, which might affect disease course.

## 1. Introduction

Prediabetes is an intermediate stage between normal glucose tolerance and type 2 diabetes mellitus (T2DM), characterized by the presence of impaired fasting glucose (IFG) and/or glucose intolerance (IGT), together with some degree of insulin resistance (IR) and pancreatic β-cell dysfunction. Since 5–10% of prediabetic people progress to overt T2DM annually, prediabetes is often considered a high-risk state for diabetes development and a key stage to implement preventive measures against its progression [1]. Prediabetes has obesity as a major risk factor and is associated with serious complications, such as hepatic steatosis, which is a major hallmark of non-alcoholic fatty liver disease (NALFD), a chronic disorder with an increased prevalence. Hepatic steatosis development and progression is driven by several pathophysiological mechanisms, including mitochondrial dysfunction, oxidative stress and impairments of autophagy and endoplasmic reticulum (ER) stress responses [2]. Noticeably, deregulated autophagy and ER stress are pathophysiological mechanisms associated with the development of IR and prediabetes as well [3].

The central risk factors for the progression of metabolic disorders, including prediabetes and hepatic steatosis, are genetic susceptibility, sedentary lifestyles and the consumption of hypercaloric diets rich in fat and/or sugar, all of which are linked to visceral adiposity, dyslipidemia, insulin resistance and T2DM [4]. Although pharmacological interventions are mandatory for T2DM management, lifestyle modifications are instead recommended as first-line interventions in stages where the disease is not yet fully established, such as in prediabetes, where subtle pathophysiological changes start to evolve in vital organs, including in the liver. Epidemiological evidences support the notion that a balanced diet, rich in fruits and vegetables, has a positive impact on metabolic health. For instance, the development of T2DM and the consumption of fruits and vegetables display an inverse relation [5,6]. Accordingly, it has been demonstrated that prediabetes progression can be slowed down or even stopped by a diet with improved quality [7]. The abundance of vegetable secondary metabolites in food matrices, namely phenolic compounds, is a well-recognized feature responsible for such health-enhancing claims. Blueberries, given their richness in polyphenols and dietary fiber, high nutritional and low caloric value, have been associated with a variety of health-related properties [8,9].

Blueberry-derived anthocyanins, flavonoids and phenolic acids can efficiently activate cellular antioxidant defenses and repress inflammatory signaling pathways. Moreover, blueberries’ prebiotic, anti-bacterial, hepatoprotective, hypoglycemic and insulin sensitizer properties have been extensively described [8,9]. A wealth of evidence outlines the beneficial effects of blueberry polyphenolic supplementation on metabolic conditions [8,9,10]. Likewise, our previous work in a prediabetic animal model demonstrated the beneficial effects of blueberry supplementation on glycemic and lipidic profile, featured by an amelioration of hepatic mitochondrial bioenergetics [9]. Still, hepatic lipid overload due to enhanced de novo lipogenesis or increased free fat acids flux from diet or peripheral tissues has also been associated with a plethora of seemingly determining factors, among them hepatic ER stress and autophagy impairment along with extra-hepatic components from distal organs, namely the gut [11,12]. Accordingly, we hypothesize whether the blueberry nutraceutical supplementation could impact hepatic autophagy and an ER stress response driven by a dietary fat overload challenge.

In this experimental work, we aim to shed light onto the impact of a blueberry nutraceutical intervention on hepatic mechanisms that govern lipid homeostasis, namely autophagy and ER stress, in prediabetes.

## 2. Materials and Methods

### 2.1. Blueberry Juice Preparation

Blueberries (Vaccinium corymbosum L. from Cultivar: Liberty) were supplied by the Cooperativa Agropecuária dos Agricultores de Mangualde (COAPE) and stored at −80 °C until processing. In order to obtain a blueberry juice (BJ) and to ensure that blueberry pulp, seeds and peel were all consumed, blueberries were weighed and blended with drinking water. To ensure that 25 g of blueberry per kg of rat body weight (BW) were consumed daily, the volume of drinking water was adjusted.

### 2.2. Blueberry Juice Characterization

BJ was characterized in terms of its phytochemical polyphenolic composition through HPLC/PDA/ESI-MSn (Supplementary Data). The BJ total phenolic content (TPC) and antioxidant capacity of BJ were also assessed.

#### 2.2.1. Phytochemical Polyphenolic Composition

Lyophilized samples were dissolved in methanol and analyzed in accordance with a previously reported method [13] with minor modifications through chromatographic analysis as previously described [14]. The identification of the main phenolic compounds was performed by a comparison of the peak areas at the maximum absorption wavelength, spectra and retention times (Supplementary Data).

#### 2.2.2. Total Phenolic Content

TPC was assessed by the Folin–Ciocalteu method adapted to a microplate reader as previously described, with minor modifications [15,16]. Succinctly, 15 μL of sample, 15 μL of Folin–Ciocalteu reagent and 150 μL of deionized water were mixed in the well, followed by the addition of 120 μL of sodium carbonate solution (700 mM). Sample absorbance was measured at 765 nm in a Synergy™ HT Multi-Detection Microplate Reader (BioTek Instruments^®^, Vermont, USA) following 1 h of incubation in the dark. Gallic acid was used as standard. Samples were determined in triplicate. Results were expressed as milligrams of gallic acid equivalents per milliliter of juice (GAE mg/mL).

#### 2.2.3. Antioxidant Capacity

Antioxidant capacity was evaluated through a 2,2′-azino-bis(3-ethylbenzothiazoline-6-sulfonic acid) (ABTS) radical scavenging capacity assay in accordance with a previously reported method [17] with minor modifications. The cation radical solution (ABTS^•+^) was obtained by adding 88 μL of potassium persulfate solution (140 mM) to 5 mL of ABTS solution (7 mM) followed by 16 h of incubation in the dark in an orbital shaker. Subsequently, the ABTS^•+^ solution was diluted until the absorbance reached 0.7 ± 0.02 at 743 nm. Then, 6 μL of various BJ dilutions were added to 300 μL of the diluted ABTS^•+^ solution in a 96-multiwell plate and after 6 min of incubation in the dark, and the absorbance (Abs) was read at 734 nm using a Synergy™ HT Multi-Detection Microplate Reader (BioTek^®^, Winooski, VT, USA). Samples were determined in triplicate. The percentage of inhibition of ABTS^•+^ was calculated using the following formula:

(1) $$ABTS\;Inhibition\left(\%\right)=\frac{\left(ABTS_{Abs}-Sample_{Abs}\right)}{ABTS_{Abs}}\times 100$$

Ascorbic acid was used as the standard. Values were plotted as a function of the concentration of ascorbic acid and the radical scavenging activity was expressed in milligrams of ascorbic acid equivalents per milliliter of juice (AAE mg/mL).

#### 2.2.4. Dietary Fiber

Total dietary fiber concentration was measured using Megazyme Test Kit (Ref: K-TDFR; Megazyme International, Bray, Ireland) and expressed in grams per 100 mg, based on the Association of Official Analytical Chemists (AOAC) method 991.43.

### 2.3. Experimental Design

Twenty-two male Wistar rats (13-week-old) were housed two per cage in the vivarium of the Coimbra Institute for Clinical and Biomedical Research (iCBR), at the Faculty of Medicine from the University of Coimbra (FMUC), under controlled environmental conditions at a temperature of 22 ± 1 °C, with 50–60% relative humidity, a 12 h light 12 h dark cycle, with tap water and food supplied ad libitum, except during fasting periods.

**Figure 1:**
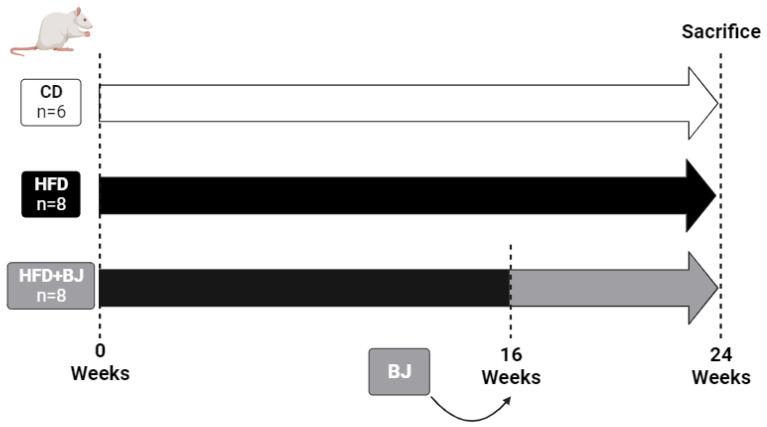
Experimental animal protocol. From weeks 16 to 24, the HFD + BJ group was daily orally supplemented with 25 g/kg of BW of blueberry juice (BJ).

After a two-week acclimation period, rats were randomly divided into three groups, according to G*Power software (version 3.1.9.7.), and assigned to the following dietary regimens, during a 24-week protocol, according to Figure 1: a group of rats under a standard control diet (CD) (4RF21, Mucedola, Milan, Italy), which provides a 3.15 kcal/g (8.6% from fat, 67.9% from carbohydrates and 23% from protein), n = 6 animals; a group of high-fat-diet (HFD)-induced prediabetes (TD. 08811, Envigo Teklad Custom Diets, West Lafayette, Indiana, EUA), which provides a 4.7 kcal/g (44.6% from fat, 40.7% from carbohydrates and 14.7% from protein), n = 8 rats; a group of rats also under HFD but supplemented with 25 g/kg of BW per day of BJ (HFD + BJ) from week 16 until the end of the protocol (24 weeks), n = 8 rats. A daily adjustment of the volume of water available in the bottle of each cage was made after the total consumption of the daily dose of BJ for the 2 animals per cage. All the animals completed the entire protocol of 24 weeks.

All animals were monitored weekly for BW and food consumption. Until week 16, the fluid (water) intake was measured twice a week, and after that point onward, the fluid (BJ) intake was measured daily. At weeks 0, 8, and 16, half of the HFD + BJ group was randomly divided (to avoid biased-metabolic outcomes) to perform a glucose tolerance test (GTT) or an insulin tolerance test (ITT), and at weeks 23 and 24, all animals performed a GTT and ITT, respectively. Experiments were conducted according to the National and European Communities Council Directives of Animal Care and the project received approval (#9/2018) by the local (iCBR) Animal Welfare Body (ORBEA) and by the National Authority (Direção Geral de Alimentação e Veterinária), on 29 October 2020.

### 2.4. Glycemic and Insulinemic Profile

#### 2.4.1. Glucose Tolerance Test (GTT) and Insulin Tolerance Test (ITT)

For ITT and GTT assays, after a 6 h fasting period, rats were injected intraperitoneally with a saline injection of insulin (0.75 U/kg BW of Novo Rapid, Novo Nordisk^®^, Paço de Arcos, Portugal), or with a glucose solution (2.0 g/kg BW, Sigma-Aldrich, Merck, St. Louis, MO, USA, EUA), respectively. Blood samples were collected from the tail vein, being the first drop of blood discarded, and the second drop used to measure baseline glucose levels. Serum glucose measurements occurred at subsequent time points following glucose or insulin administration (ITT assay: 15, 30, 45, 60, 120 min; GTT assay: 15, 30, 60, 120 min) at selected time-points. The glucose levels were measured using a portable commercial glucometer kit (GlucoMen^®^ aero 2K, A. Menarini Diagnósticos, Paço de Arcos, Portugal). The area under curve (AUC) of ITT and of GTT curves were calculated using the trapezoidal method [18,19].

#### 2.4.2. Fasting and Postprandial Glucose and Insulin

Blood samples used to determine the fasting glucose and insulin levels were collected on the day of the ITT assay (week 24) at the 0 min time point, while postprandial glucose and insulin levels were determined the day before animal euthanasia using capillary tubes (Microvette^®^ CB 300Z, Sarstedt AG & Co., Nümbrecht, Germany). Blood samples were centrifuged at 1500× *g* for 10 min at 4 °C for insulin and serum determinations. Serum insulin levels were quantified using a rat insulin enzyme-linked immuno-sorbent assay (ELISA) kit (Mercodia, Uppsala, Sweden) according to the manufacturer’s kit procedure. Serum glucose levels were measured using a portable commercial glucometer kit (GlucoMen^®^ aero 2K, A. MENARINI diagnostics).

### 2.5. Blood and Tissue Collection

At week 24, animals were anaesthetized in a chamber saturated with isoflurane (IsoFlo^®^, Abbott Laboratórios, Lda., Amadora, Portugal) followed by intraperitoneal injection of 150 mg/kg of ketamine chloride (1 g/mL; Imalgene^®^) in chlorpromazine 2.5% (Largactil^®^). Blood was immediately collected through heart puncture, centrifuged at 3500 rpm for 15 min (4 °C) and stored at −20 °C. Upon sacrifice, rats were transcardially perfused with ice-cold PBS and epididymal white adipose tissue (eWAT), whilst the interscapular brown adipose tissue (iBAT) and liver were isolated, washed and weighed. Samples were divided into 3 sections for distinct purposes: a first section was kept in a neutral buffered formalin solution to be used for histological analysis; for fluorescence microscopy, samples were placed in OCT CryoMatrix (6769006, Thermo Fisher Scientific, Waltham, MA, USA); for protein analysis, samples were snap frozen in liquid nitrogen. Samples were stored at −80 °C for later analysis.

### 2.6. Serum Lipid Profile

Serum levels of triglycerides (TGs), low-density lipoprotein cholesterol (LDL-c), high-density lipoprotein cholesterol (HDL-c), and the total cholesterol was measured as previously described [18] through automatically validated methods using a Chemistry Analyzer (Hitachi 717 analyzer, Roche Diagnostics GMBH, Mannheim, Germany).

### 2.7. Extraction and Quantification of Gut Microbiota in Feces

#### 2.7.1. DNA Extraction from Stool

Genomic DNA was extracted and purified from fecal samples using NZY Tissue gDNA Isolation Kit (Nzytech^®^, Ref. MB135, Lisboa, Portugal) with few modifications [20]. Between 170 and 200 mg of feces were homogenized in TE buffer (10 mM Tris/HCl; 1 mM EDTA, pH 8.0) and centrifuged for 15 min at 4000× *g*. After the supernatant was discarded, the pellet was resuspended in 1 mL of buffer NT1. Then, 25 μL of proteinase K was added to 200 μL of supernatant and incubated for 1 h and 45 min at 56 °C. The remaining steps followed the manufacturer’s instructions. DNA quantification and purity were assessed using a NanoDrop spectrophotometer (Thermo Fisher Scientific, Waltham, MA, USA).

#### 2.7.2. Real-Time PCR for Microbial Analysis of Stool

Real-time PCR was performed using a CFX96 Connect Real-time PCR System instrument and an iQ™ SYBR^®^ Green Supermix in sealed 96-well microplates, from Bio-Rad Laboratories (Hercules, CA, USA). PCR reaction mixtures were composed of 1 μL of DNA (equilibrated to 20 ng), 1 μL of each primer (final concentration of 0.1 μM), 5 μL of iQ™ SYBR^®^ Green and 2 μL of water. The conditions for PCR amplifications and the primer sequences (STAB VIDA, Lisboa, Portugal) used to target the 16S rRNA gene of the bacteria are listed in Table 1.

Data were processed and analyzed using the CFX Maestro software (Bio-Rad Laboratories (Hercules, CA, USA) and standard curves were constructed using serial tenfold dilutions of bacterial genomic DNA, according to the following webpage http://cels.uri.edu/gsc/cndna.html (accessed on 20 May 2023). Bacterial genomic DNA used as a standard (Table 1) was obtained from DSMZ (Braunschweig, Germany). The copy number of the 16S rRNA gene for each bacterial strain used as a standard and genome size was obtained from the NCBI Genome database (www.ncbi.nlm.nih.gov (accessed on 20 May 2023)). Data are presented as the mean values of the duplicate PCR analysis. The *Firmicutes/Bacteroidetes* ratio was obtained by dividing the number of copies of the *Firmicutes* division by the number of copies of *Bacteroidetes* division.

### 2.8. Fecal Short-Chain Fatty Acids Determination

Stool samples were defrosted and homogenized. Short-chain fatty acids (SCFAs) were extracted with diethyl ether and quantified by gas chromatography as previously described [25]. Acid concentrations were expressed in micromoles per gram (μmol/g).

### 2.9. Liver, eWAT and iBAT Histomorphology

#### 2.9.1. Hematoxylin and Eosin (H&E) Staining

Liver, eWAT and iBAT samples were formalin-fixed, embedded in paraffin wax and immersed in hematoxylin stain solution, as previously described [14].

#### 2.9.2. Image Analysis and Data Quantification

##### Liver

Two high-resolution images per animal, with 5–7 animals per group and imaged at 40-fold magnification were captured under the same parameter settings for hepatocyte morphometry analysis using a Zeiss microscope Mod. Axioplan 332 2 (Zeiss, Jena, Germany). To evaluate the liver morphology and the degree of steatosis, approximately 200 hepatocytes per animal were analyzed using the FIJI (ImageJ v2) plugin Cell Counter.

Each acquired image was imported to ImageJ and analyzed individually. The number of total hepatocytes in each image was calculated by manually counting all the visible hepatocyte nuclei.

To assess the total steatosis score (%), the ratio between the total number of steatotic hepatocytes and the total number of hepatocytes times 100 were calculated.

(2) $$Total\;Steatosis\;Score\left(\%\right)=\frac{Number\;of\;steatotic\;hepatocytes}{Total\;number\;of\;hepatocytes}\times 100$$

##### eWAT

Between 5 and 10 high-resolution images per animal, with 5–8 animals per group, were captured by an Axiocam 105 color (Zeiss, Jenna, Germany) camera under the same parameter settings for adipocyte morphometry analysis, using a Zeiss microscope Mod. Axioplan 332 2 (Zeiss, Jenna, Germany). Slides were imaged at 10-fold magnification. To quantify the mean adipocyte area, mean adipocyte diameter, adipocyte area distribution and adipocyte diameter distribution, an average of 512 adipocytes per animal were analyzed using the FIJI (ImageJ v2) plugin Adiposoft. Images were captured and imported into ImageJ software and analyzed by the ImageJ plugin Adiposoft. To ensure that only adipocytes were counted, each image was manually inspected. The artifacts and broken adipocytes unduly analyzed and accounted by the software were recorded and manually eliminated from the output-excel sheet that contained the diameter and area data of all the adipocytes in each image. The mean adipocyte area and mean adipocyte diameter were calculated for each animal and for each group.

##### iBAT

Five high-resolution images per animal (5–8 animals per group) were captured by Axiocam 105 Color (Zeiss, Jenna, Germany) camera under the same parameter settings for adipocyte morphometry analysis using a Zeiss microscope Mod. Axioplan 332 2 (Zeiss, Jenna, Germany). Slides were imaged at 20-fold magnification. FIJI (ImageJ v2) plugin Adiposoft was used to quantify the mean lipid droplet area and distribution. iBAT images were captured and imported into ImageJ software as described in the eWAT section. Similarly, iBAT images were analyzed by the ImageJ plugin Adiposoft. The mean lipid droplet area was calculated for each animal and for each group.

### 2.10. Hepatic Triglycerides Quantification

The liver samples TGs content was measured by an enzymatic colorimetric assay, as previously described [14].

### 2.11. Hepatic Enzymes Quantification

Serum alanine aminotransferase (ALT) and aspartate aminotransferase (AST) concentrations were determined as previously described in [19], through automatic validation methods and equipment (Hitachi 717 analyzer, Roche Diagnostics GMBH, Mannheim, Germany).

### 2.12. Protein Expression by Western Blotting

#### 2.12.1. Protein Extraction and Quantification

Approximately 100 mg of frozen iBAT and 50 mg of liver were homogenized by mechanical dissociation using a handheld tissue homogenizer in a cold (4 °C) radioimmunoprecipitation assay (RIPA) lysis buffer containing 50 mM Tris-HCl (pH 8.0), 150 mM NaCl, 0.1% (*v*/*v*) sodium dodecyl sulphate (SDS), 1% (*v*/*v*) Triton X-100, 5 mM ethylenediaminetetraacetic acid (EDTA), supplemented with 1 pill of protease inhibitor cocktail (11836170001, Roche Diagnostics GmbH, Germany), 1 mM of phenylmethylsulfonyl fluoride (PMSF) and 1 mM of sodium orthovanadate (Na_3_VO_4_). iBAT and liver homogenates were left on ice for 1 h, and vortexed every 15 min. iBAT sample lysates were centrifuged for 15 min at 15,000 rpm at 4 °C and liver sample lysates were centrifuged for 20 min at 13,000 rpm at 4 °C. After centrifugation, the supernatant fractions were collected and centrifuged again. This process was repeated three times in iBAT samples and twice in liver samples. After the last centrifugation, the supernatant fractions were collected and the protein lysates of each sample were used to determine the protein concentration. The remaining samples were stored at −80 °C.

The protein concentration was determined using the bicinchoninic acid (BCA) assay (Pierce™ BCA Protein Assay Kit, Pierce Biotechnology, Rockfor, IL, USA) using bovine albumin serum (BSA) standard solutions. Absorbance reading was performed at 570 nm using BioTek Synergy™ HT and Gen5 Data Analysis Software after the plate was incubated for 30 min at 37 °C. The total protein concentration in each sample was calculated considering the calibration curve obtained through the BCA assay.

#### 2.12.2. Polyacrylamide Gel Electrophoresis and Immunodetection

iBAT and liver protein samples were denatured with sample buffer (6-fold) (0.35 M Tris-HCl (pH 6.8), 30% (*v*/*v*) glycerol, 0.65 M dithiothreitol (DTT), 10% (*w*/*v*) SDS, 0.03% (*w*/*v*) bromophenol blue) for 5 min at 95 °C.

For the Western blot analysis, 10 μg of iBAT protein and 30 μg of liver protein were loaded per lane and separated by electrophoresis on a 10–15% SDS–polyacrylamide gel electrophoresis (SDS-PAGE) in running buffer (125 mM Tris-base, 950 mM glycine and 0.5% (*w*/*v*) SDS, pH 8.3) at 70 volts for 20 min and then at 100 volts for 150–210 min until the prestained marker (A8889.0500 Protein Marker VI (10-245), AppliChem GMbH, Darmstadt, Germany) reached the desired level depending on the molecular weight of the target protein, at 4 °C. After electrophoresis and activation of 0.45 μm polyvinylidene difluoride (PVDF) membranes (Amersham™ Hybond™, GE Healthcare, Buckinghamshire, UK), proteins were electro-transferred in transfer buffer (100 mM *N*-cyclohexyl-3-aminopropanesulfonic acid (CAPS), pH 11) at 100 volts, for 90 min at 4 °C. Following protein transfer, Ponceau S staining was performed to assess the transfer quality and efficiency and to confirm an equal amount of protein in each well. Distaining was performed with 0.1 M NaOH and Tris-buffered saline (150 mM NaCl, 20 mM Tris-HCl, pH 7.6) containing 0.1% (*v*/*v*) Tween-20 (TBS-T) wash. Thereafter, membranes were blocked in non-fat dry milk in TBS-T or bovine serum albumin (BSA) in TBS-T (Table 2), for 1 h with agitation, at room temperature. Membranes were then incubated with primary antibodies (Table 2), overnight at 4 °C with agitation. After incubation, membranes were washed with TBS-T and incubated with adequate secondary antibodies (Table 2) for 1 h at room temperature with agitation. After secondary antibody incubation, membranes were washed again with TBS-T. The intensity of the bands was detected by enhanced chemiluminescence substrate (ECL) (R-03031-D25, R-03025-D25 WesternBright™ ECL and Peroxide, Advansta, San Jose, CA, USA) and (R-03027-C50, R-03025-C50, WesternBright™ Sirius and Peroxide, advansta, USA) in the ImageQuant™ LAS500 (GE Healthcare, Buckinghamshire, UK). To ensure equal protein loading, membranes were re-incubated with antibodies against housekeeping proteins, including β-actin, glyceraldehyde 3-phosphate dehydrogenase (GAPDH), and calnexin (Table 2). Results were normalized against these loading proteins and expressed as a percent of control. Optical density of the bands was quantified by densitometry, using Image J Software (LOCI, University of Wisconsin, USA).

### 2.13. Data Processing and Statistical Analysis

Results were expressed as mean ± standard error of the mean (SEM) using GraphPad Prism software, version 6.01 (GraphPad Software, Inc., La Jolla, CA, USA). Differences were considered statistically significant at *p* values < 0.05. Differences between experimental groups were compared using the nonparametric Kruskal–Wallis test (followed by Dunn’s test for multiple comparisons) for non-normally distributed data or the parametric test one-way ANOVA, followed by Tukey’s test for multiple comparisons) for normally distributed data. Repeated measures ANOVA, followed by Tukey’s post hoc test, were used to compare the glucose levels throughout the GTT and ITT assays and BW evolution during the experimental period.

## 3. Results

### 3.1. Blueberry Juice Composition Characterization

Many beneficial health-related properties of blueberries, namely their prebiotic and antioxidant potential, have been associated with the abundance of dietary fiber and phenolic compounds, respectively [26]. Accordingly, we quantified the dietary fiber (Total: 8.080%, insoluble: 7.865% and soluble: 0.215%) and total phenolic content (0.574 mg of GAE/mL of juice) and antioxidant capacity (0.336 mg of AAE/mL of juice) along with the characterization of the main polyphenols present in BJ. Our results confirm that BJ obtained from *Vaccinium corymbosum* L. (Cultivar: Liberty) is enriched in both dietary fiber and polyphenols. BJ chromatographic profile further revealed that flavonoids (e.g., isorhamnetin-hexose-deoxyhexoside, laricitrin-O-pentoside, quercetin-O-hexoside, quercetin-O-pentoside and quercetin-O-deoxyhexoside), hydroxycinnamic acids (e.g., sinapic, caffeic and ferulic acid derivatives, chlorogenic acid and caffeoyl-hidroxydihidro-CQA derivative) and anthocyanins (e.g., delphidin, malvidin) are the predominant polyphenols (Supplementary Data: Figures S1 and S2, Table S1).

### 3.2. Effects of BJ Nutraceutical Intervention on Glycemic and Insulinemic Profile

**Figure 2:**
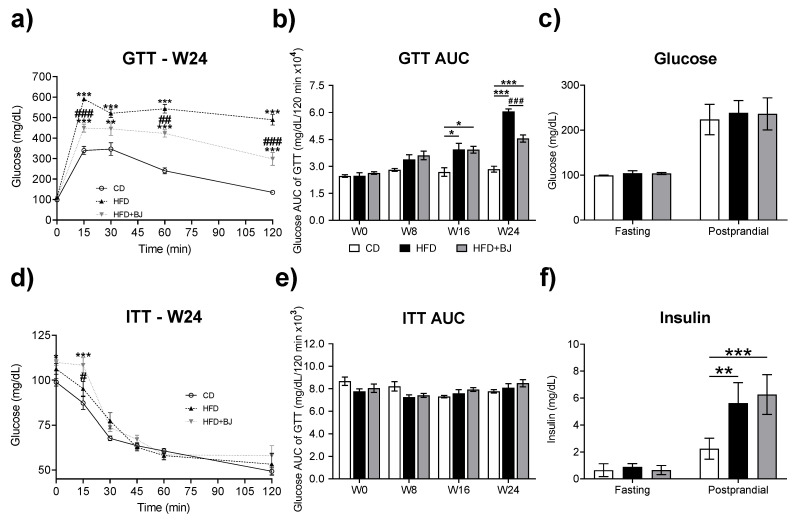
Glycemic and insulinemic profile. (**a**) Changes in blood glucose levels at different time points (glucose tolerance test (GTT) assay) at week 24 expressed as milligrams of insulin per deciliter of blood. (**b**) Area under the curve (AUC) of the GTT assay, expressed in milligrams per deciliter per 120 min times 104. (**c**) Fasting and postprandial serum glucose levels expressed as milligrams per deciliter. (**d**) Changes in blood glucose levels at different time points (insulin tolerance test (ITT) assay) at week 24 expressed as milligrams of glucose per deciliter of blood. (**e**) AUC of ITT assay, expressed as milligrams per deciliter per 120 min times 103. (**f**) Fasting and postprandial serum insulin levels expressed as milligrams per deciliter. CD—control diet; HFD—high-fat diet; HFD + BJ—high-fat diet supplemented with blueberry juice; data are expressed as mean ± SEM (n = 4–8 per group). * *p* < 0.05, ** *p* < 0.01, *** *p* < 0.001 vs. CD; # *p* < 0.05, ## *p* < 0.01 and ### *p* < 0.001 vs. HFD, using a one-way ANOVA followed by a Tukey multiple comparison test.

To determine when animals exhibited a phenotype consistent with prediabetes and to evaluate the impact of BJ supplementation on glucose tolerance and insulin sensitivity, we monitored prediabetic rats glycemic (GTT) and insulinemic (ITT) profile every 8 weeks, over the 24-week study period, and assessed fasting and postprandial glucose and insulin levels at the end of the study (Figure 2).

We found that, at week 16, the two groups of animals under HFD (HDF and HFD + BJ) displayed glucose intolerance (Figure 2b), a main feature of prediabetes. Thus, this time-point was elected to introduce blueberry-nutraceutical intervention. At the end of the study, the HFD + BJ group displayed a statistically significant (*p* < 0.001) reduction in the GTT AUC in comparison with the HFD group (Figure 2a,b), along with normal levels of fasting and postprandial glycaemia when compared to the HFD group (Figure 2c). Both the HFD and HFD + BJ groups displayed elevated postprandial insulin (*p* < 0.01; *p* < 0.001) (Figure 2f), which might reflect the attempt to overcome an increment in blood glucose (compensatory hyperinsulinemia), a feature of the prediabetic state [27,28].

### 3.3. Effects of BJ Nutraceutical Intervention on Body and Tissue Weights

To assess whether BJ supplementation could afford protection against HFD-induced body weight gain, we monitored the rats’ BW gain and determined liver, eWAT and iBAT depots relative weight (Table 3).

Our results clearly indicate that BJ was unable to correct the BW gain (*p* < 0.05) or increased liver (*p* < 0.01), eWAT (*p* < 0.001) and iBAT (*p* < 0.001) tissue weights. To further monitor rats’ metabolic profile, food and beverage intake were measured weekly throughout the 24-week study period. At week 24, we observed a decreased food intake in both HFD and HFD + BJ-fed groups when compared to the CD-fed group (*p* < 0.0001). With respect to the beverage intake, our measurements demonstrated a significant decrease in the HFD group when compared to the control (*p* < 0.01) and an increase in the group supplemented with BJ (*p* < 0.0001), possibly due to their pleasant organoleptic features (Table 3).

Caloric intake driven from each macronutrient and total caloric intake were calculated based on the diet and BJ composition and food and beverage intake. As expected, when compared to the CD-fed group, both the HFD and HFD + BJ groups exhibited a notably reduced higher caloric intake driven from the lipids (*p* < 0.0001) and a significantly lower caloric intake driven from both carbohydrates (*p* < 0.0001) and proteins (*p* < 0.0001) (Table 3). However, no statistical differences were found in the total caloric intake.

### 3.4. Effects of BJ Nutraceutical Intervention on Gut Microbiota

To further characterize the impact of BJ supplementation on HFD-induced dysbiosis, we analyzed the fecal microbiota composition (Table 4).

BJ intervention corrected the relative over-abundance of *Firmicutes* (*p* < 0.01) and the *Firmicutes/Bacteroidetes* ratio (*p* < 0.001) driven by HFD feeding. Still, this nutraceutical intervention failed to improve the *Bifidobacterium* spp., and *Lactobacillus* spp. decreased the relative abundance observed upon HFD feeding (*p* < 0.01; *p* < 0.05). Next, we analyzed the fecal SCFA content as a readout of gut microbial dietary fiber fermentation given their well-established benefits in metabolic impairments, namely prediabetes [29,30,31]. Aligned with our previous findings [26], we found a significant decrease in the fecal contents of C2 acetic, iC5 isovaleric and C6 caproic acids in HFD-fed animals (*p* < 0.05; *p* < 0.05; *p* < 0.01, respectively) which was further aggravated in the HFD + BJ-fed group (*p* < 0.05; *p* < 0.01; *p* < 0.01), regardless of the blueberries’ richness in dietary fiber (Table 4).

### 3.5. Effects of BJ Nutraceutical Intervention on Adiposity

**Figure 3:**
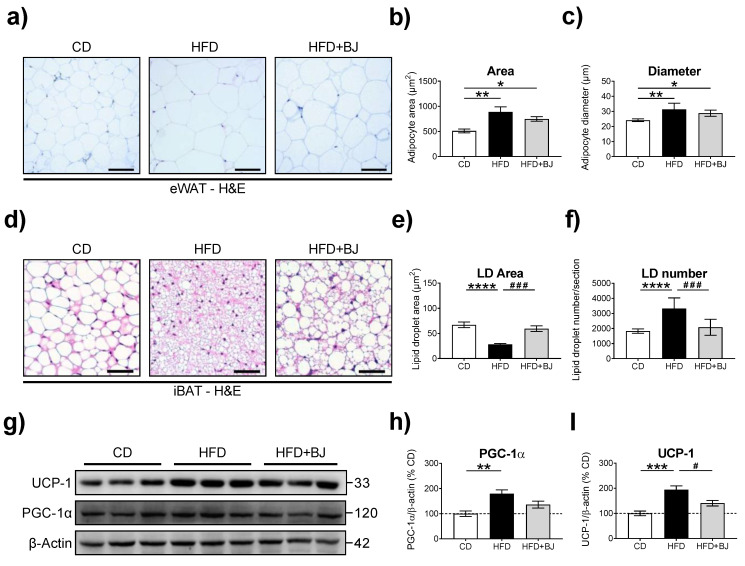
Analysis of epididymal white adipose tissue (eWAT) and interscapular brown adipose tissue (iBAT) adipocyte morphology and dimensions, and an assessment of the thermogenic marker in iBAT. (**a**) Representative images of hematoxylin and eosin (H&E) staining of eWAT sections showing adipocytes at 10× magnification (scale bar = 100 μm). (**b**) Mean adipocyte area (μm^2^) and (**c**) mean adipocyte diameter (μm) in eWAT sections. (**d**) Representative images of H&E staining of iBAT sections showing adipocyte lipid droplets (LD) at 20× magnification (scale bar = 50 μm). (**e**) Mean LD number per section. (**f**) Mean LD area (μm^2^). (**g**) Immunoblotting of thermogenic markers uncoupling protein-1 (UCP-1) and peroxisome proliferator-activated receptor-gamma coactivator-1alpha (PGC-1α), and their (**h**,**i**) corresponding protein levels in iBAT protein extracts from male Wistar rats fed with a CD and HFD diet for 24 weeks and an HFD diet supplemented with BJ for 8 weeks. Data are means ± SEM (n = 5–8 per group); * *p* < 0.05, ** *p* < 0.01, *** *p* < 0.001 and **** *p* < 0.0001 vs. CD; # *p* < 0.01 and ### *p* < 0.001 vs. HFD, using a one-way ANOVA followed by a Tukey multiple comparison test.

Given that BJ supplementation failed to counteract HFD-elicited BW gain and that adipose tissue expansion may result from increased adipocyte number (hyperplasia) and/or adipocyte size (hypertrophy), we proceeded to the histological characterization of adipocyte size/diameter in eWAT and iBAT resorting to H&E staining (Figure 3).

We found no differences in eWAT adipocyte size between the HFD and HFD + BJ groups, although both groups displayed increased adipocyte dimensions in comparison to the CD-fed group (Figure 3a). To further explore these differences, we performed a quantitative histological analysis by assessing the cell size parameters (area and diameter) (Figure 3b,c). Both groups presented a significant increase in the eWAT mean adipocyte area and diameter when compared to the CD group.

While the major function of WAT is energy storage, BAT is primarily involved in energy expenditure, namely through the activation of thermogenic pathways. Given the positive energy balance observed upon HFD feeding, we then asked whether BJ could impact the iBAT lipid management through the examination of the lipid droplet size/number and key thermogenic markers. The HFD-fed group displayed a reduced area and increased number of lipid droplets (*p* < 0.0001) alongside increased iBAT thermogenesis, assessed by PGC-1α and UCP-1 protein levels (*p* < 0.01; *p* < 0.001). Unexpectedly, BJ supplementation arrested iBAT thermogenesis and energy expenditure, confirmed by the enlarged lipid droplets observed in histologic evaluation (Figure 3d–f).

### 3.6. Effects of BJ Nutraceutical Intervention on Hepatic Lipid Management

**Figure 4:**
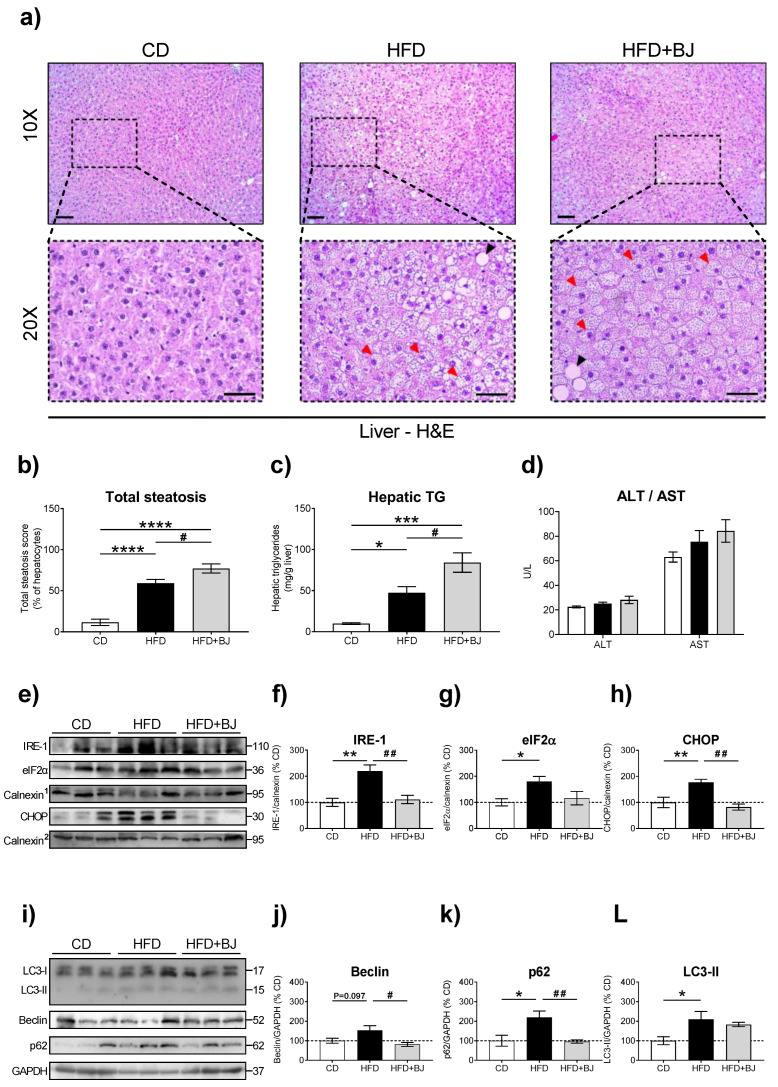
Histological and quantitative assessment of hepatic steatosis and autophagy and endoplasmic reticulum stress markers quantification. (**a**) Representative images of hematoxylin and eosin (H&E) staining of liver sections from rats after 24 weeks of different dietary regimens and 8-week BJ ingestion showing normal (nonsteatotic), microsteatotic (red arrows) and macrosteatotic (black arrows) hepatocytes at 10× and 20× magnification (scale bar = 100 μm). (**b**) The total steatosis score expressed as a percentage of hepatocytes. (**c**) Hepatic triglyceride (TG) content expressed as milligrams per grams of liver. (**d**) Plasma levels (U/L) of alanine transaminase (ALT) and aspartate transaminase (AST). (**e**) Representative Western blot images of ER stress markers IRE-1, eIF2α and CHOP, and their (**f**–**h**) corresponding protein levels. (**i**) Representative Western blot images of autophagy markers LC3-II, Beclin and p62, and (**j**–**l**) corresponding to the protein levels in liver protein extracts from rats fed with CD, HFD diet and HFD diet supplemented with BJ for 8 weeks. Data are presented as mean ± SEM (n = 6–8 rats per group); * *p* < 0.05, ** *p* < 0.01, *** *p* < 0.001 and **** *p* < 0.0001 vs. CD; # *p* < 0.05 and ## *p* < 0.01 vs. HFD, using a one-way ANOVA followed by a Tukey multiple-comparison test.

Impairments on glycemic and lipidic profiles often co-exist in prediabetic conditions [4]. Accordingly, we intended to characterize the effects of BJ intervention on lipid management once challenged by a dietary fat overload (Table 5 and Figure 4).

Unexpectedly, the BJ worsened the serum TGs (*p* < 0.05 versus HFD) and total cholesterol (*p* < 0.05) contents evoked by HFD feeding (Table 5). Likewise, it aggravated the percentage of steatotic hepatocytes (Figure 4a–c) without major changes in plasmatic ALT/AST levels, two common markers of liver injury (Figure 4d). To obtain an in-depth knowledge on the cellular mechanisms governing the exacerbated lipotoxicity driven by BJ supplementation, we focused our attention on the autophagy and ER stress response given their chief roles on hepatic triglyceride storage and lipid overload management. We found increased protein levels of well-known ER stress (IRE-1: *p* < 0.01; elF2α: *p* < 0.05; CHOP: *p* < 0.01; Figure 4e–h) and autophagy (LC3-II: *p* < 0.05; p62: *p* < 0.05; Beclin: *p* = 0.097; Figure 4i–l) markers upon HFD feeding. Again, BJ supplementation arrested the autophagic and ER stress responses, constituting key mechanisms to counteract dietary lipid overload.

## 4. Discussion

Blueberry juice supplementation between weeks 16 and 24 was able to ameliorate glucose intolerance in the prediabetic animals, which agrees with previous studies [9,27,32,33]. This effect could be attributed to the previously reported blueberries’ capacity to exert insulin-sensitizing, antioxidant, inflammatory and prebiotic properties, acting in distinct metabolic tissues, including the pancreas, the liver and the adipocyte tissue, as well as the gut, as previously reviewed [9]. However, BJ was unable to counteract an HFD-induced increase in BW and adiposity. A previous animal study showed a decrease in diet-induced BW gain and adiposity in mice treated with blueberry extract [34] while another one reported no effects of blueberry powder in HFD-fed mice [32]. Our observations are in line with the former study and consistent with previous findings from our group in animals supplemented with high-sugar diet [26].

BAT is recognized as the major site for non-shivering thermogenesis, controlling whole-body energy expenditure and body fat due to the abundance of mitochondria with a high expression of UCP-1 [35,36]. It was hypothesized that Wistar rats first increase lipid storage in peripheral WAT and subsequently overexpress UCP-1-related thermogenesis markers in BAT as an adaptive process in response to excess lipid consumption [35]. Considering that thermogenesis roughly contributes to 15–20% of the total energy expenditure in rodents, the impact of this mechanism on energy balance and BW regulation is significant [37]. Interestingly, BJ supplementation decreased the expression of thermogenic markers along with an increase in brown adipocyte lipid droplet size, indicating a decrease in mitochondrial oxidation of the lipidic content present in BAT adipocytes [38]. In a previous work using healthy Wistar rats, we observed that a long-term BJ intake triggered a mitochondrial-adaptative setting, featured by an accentuated bioenergetic remodeling in isolated hepatic mitochondria [14]. A similar impact of BJ on the iBAT mitochondrial function could be hypothesized, causing thermogenesis arrest, thus contributing to the positive energy balance reflected by an increase in BW gain and adiposity. Unexpectedly, but in agreement with these effects, the BJ supplementation aggravated the HFD-induced lipotoxicity, as viewed by the worsening of serum hypertriglyceridemia and hepatic steatosis, together with the inhibition of hepatic autophagic and ER stress responses.

Several studies have reported the positive effects of blueberry-enriched diets on dyslipidemia [26,39,40,41], describing a reduction in TC and LDL-c levels in obese Zucker rats supplemented with 8% wild blueberry during 8 weeks [41] and with 2% blueberry powder for 13 weeks [40], and a decrease in TC and LDL-c contents in Wistar rats under BJ treatment for 14 weeks [26]. In contrast with those findings, we observed that BJ not only failed to attenuate the HFD-induced increase in LDL-c levels, but also further increased the serum TC and TGs levels. Considering that thermogenic activation may result in fat oxidation, contributing to liver protection [35], and that increased BAT activity promotes the clearance of TGs, the decrease in thermogenic activation observed in the BJ-treated animals may contribute to explaining the increase in serum and hepatic TGs. These results contrast with previous studies showing the efficacy of a wide variety of polyphenols in inducing thermogenesis and fatty acid oxidation [37]. For instance, a study using a phenolic blueberry extract in mice reported an improvement in genetically and diet-induced metabolic syndrome, which was linked to improved hepatic lipid metabolism and increased energy expenditure in BAT [34]. Nonetheless, despite the widely acknowledged antioxidant properties attributed to polyphenols, several preclinical and clinical studies have reported detrimental effects arising from the overconsumption of dietary polyphenols [42,43,44]. Depending on the dose, the disruption on redox homeostasis can indeed cause hepatotoxicity [42,45]. For instance, the excessive intake of flavan-3-ols from *Camellia sinensis* teas can promote the collapse of the mitochondrial membrane potential, inducing pro-oxidant effects resulting in hepatotoxicity [46], and even a low dose (150 mg/Kg) of epigallocatechin gallate (EGCG) was associated with hepatotoxicity in rodents [45]. Thus, considering the detrimental effects of BJ on healthy Wistar rats’ hepatic mitochondrial function [14], it is reasonable to suggest that the same dose of BJ (25 g/kg BW/day) may have also had deleterious effects on the hepatic mitochondrial function of our prediabetic animals, possibly compromising fat oxidation and further contributing to hepatic steatosis.

Hepatic lipid overload due to enhanced de novo lipogenesis or increased free fat acids flux from diet or peripheral tissues has been associated with impaired ER stress and autophagy in metabolic disorders, including in prediabetic and diabetic animals [11,47,48,49,50,51]. Despite the existence of a few studies on the effects of polyphenol/anthocyanins extracts on hepatic ER stress response and autophagy, to the best of our knowledge, there are no studies regarding the effects of whole blueberry supplementation on these mechanisms in the liver of prediabetic animals. Here, we found that BJ supplementation decreased the expression of the ER stress markers IRE-1 and CHOP, suggesting a downregulation of the IRE-1 and PERK pathways. In contrast to our results, another study showed that malvidin, a blueberry-containing polyphenol, upregulated the CHOP and BiP protein expression in activated rat hepatic stellate T6 cells (HSC-T6), promoting apoptosis and hepatoprotection [52]. On the other hand, in line with our results, diabetic Wistar rats treated with morin polyphenol, exhibited a downregulation of PERK and IRE-1 response pathways, revealed by the decrease in BiP, IRE-1, XBP-1 and CHOP (p-PERK/PERK) protein expression [51]. Similarly, Kandeil et al. (2019) also reported a decrease in the BiP, XBP-1 and CHOP gene expression in HFD-fed Wistar rats supplemented with the *Zingiber officinalis* polyphenol extract [53]. Regarding autophagy, the observed decrease in the Beclin and p62 expression and the trend towards the decrease in LC3-II expression found in the BJ-supplemented group of our study suggests that both the nucleation and elongation phases of autophagy are downregulated. Accordingly, the autophagy impairment is associated with a reduction in the lipid stores/droplets breakdown, resulting in the accumulation of hepatic TG [11]. Hence, the arrest in autophagy and the decrease in ER stress response observed in the BJ-supplemented prediabetic animals were two main contributors to the increase in serum and hepatic TGs’ content. In other studies, supplementation with blueberry-containing polyphenols was associated with beneficial hepatic effects through autophagy induction. For instance, Zhuge et al. (2020) reported that the supplementation with the blueberry polyphenol extract in a mouse model of NAFLD improved hepatic steatosis by lowering the hepatic TG content, mentioning a decrease in p62 and an enhancement of LC3-II/LC3-I proportion in the liver tissue [54]. Similarly, the downregulation of p62 and the increase in LC3-II and Beclin were also associated with autophagy induction in mice supplemented with pterostilbene, an active constituent of blueberries [55]. In contrast with these results, another study reported a reduction in fatty lipid droplets in the liver of high-fat–high-sugar diet-fed prediabetic Sprague Dawley rats treated with the polyphenol TSG (2,3,5,4′-tetrahydroxystilbene-2-O-β-D-glucoside). Intriguingly, this study also noted an increase in the protein expression of Beclin, p62 and LC3-II, suggesting that TSG prompted liver autophagic cell death as a protective response against the prediabetic challenge [49].

Red fruits like the blueberry, known for their high levels of both polyphenols and fiber content, have been associated with prebiotic activity, modulating gut microbial community [9,56]. Intestinal microbiota plays a major role in the host metabolism, regulating, among other players, the production of SCFAs, which protect the gut barrier, exert anti-inflammatory effects and modulate energy and lipid metabolism, acting on major peripheral (liver, muscle and adipocyte tissue) and central (brain) insulin sensitizing tissues [57,58]. Impaired gut microbiota community and/or diversity (dysbiosis) has been linked with the development of metabolic disorders, including prediabetes and diabetes [59]. In addition, a reduction in SCFAs has been correlated to lipid dysmetabolism and hepatic steatosis [60,61]. SCFA-treated rats presented decreased plasma and hepatic TGs and cholesterol levels [62] as well as an increased UCP-2 expression, resulting in the stimulation of fat oxidative metabolism in the liver and adipose tissue [12]. In the present study, using 16S mRNA quantification by RT-PCR, no major changes were obtained on the fecal microbial species evaluated in the group under BJ supplementation versus the HFD-fed animals, despite the reduction in the *firmicutes* and *firmicutes/bacteroidetes* ratio. However, there was a trend in the reductions in all SCFAs, which is in agreement with the previous study from our group [26]. So, considering the major role of SCFAs in the regulation of lipid metabolism, including in the liver by activating AMPK activity, increasing insulin sensitivity and reducing lipid accumulation [57], the impact of BJ on the fecal SCFA content in the HFD-fed animals may also explain the increased lipid deposition in the hepatic tissue, which deserves further clarification. Moreover, future research should make use of metagenomic sequencing technology to access the effects of BJ on gut microbial species, thus enhancing the data robustness regarding the functional diversity of the bacterial community.

We previously observed that the same dose of BJ did not affect the glycemic profile in healthy Wistar rats [14], but ameliorated the glycemic profile of prediabetic rats in the present protocol, in agreement with another previous study from us [26]. Interestingly, while the lipid profile remained unaffected in a healthy condition [14], BJ had a beneficial effect in prediabetic rats with a more aggravated lipid profile than those used in the present study [26], whereas in this study, it promoted its worsening. The discrepancy of effects could be eventually explained by several factors, including the features related with the models of disease used, as well as a diversity of factors associated with blueberry composition and properties. Blueberries’ complex phenolic composition and TPC are highly influenced by numerous factors, such as the blueberry strain, plant genetics, cultivation methods, agronomic conditions, environmental factors as well as the extent and type of post-harvest processing and storage [63,64,65]. These factors establish a high variability of polyphenolic profiles and a wide range of TPC. For instance, while Yousef et al. (2013) showed blueberry TPCs ranging from 1.63 to 2.87 mg (GAE)/g [66], Weaver et al. (2023) reported that the weight of blueberry powder needed to achieve the same dose of TPC (25 mg/kg BW) varied from 189 to 285 mg [67]. Additionally, it has been shown that the bioavailability of polyphenolic compounds in rats was not predicted by the TPC [67], further increasing the complexity of predicting the dose-biological response relationship. An example is the impact of blueberry on rat gut microbiome diversity, which seems to be dose-dependent [67]. It has been acknowledged that dietary phytochemicals can act as hormetins [68]. A hormetic response occurs when a mild stress stimulus triggers adaptations that will enhance resistance to a more severe stress condition, which can arise, for example, from higher doses of the same stressor or from other less-specific stressors, such as thermal, oxidative and metabolic stressors [69]. This concept has been used to characterize the phenomenon in which a specific condition or substance is able to induce biological opposing effects at different doses and conditions [70]. Our data support this concept for blueberry juice since a distinctive impact on the liver lipid metabolism was observed under different conditions, despite identical benefits on glucose tolerance.

This study serves to highlight the considerable variability in the individuals’ response to the consumption of this type of phenolic-rich foods, emphasizing the influence of several factors, including the dose as well as the individual consumer characteristics. In the case of prediabetic people, it is known that practicing a balanced diet is important to avoid disease progression; however, as previously mentioned for the impact of pharmacotherapy, good glycemic control per se is not enough to modify the course of the disease, and further attention should be directed towards understanding the impact on other mechanisms at the cellular and molecular levels.

## 5. Conclusions

In conclusion, despite the improvement in glucose tolerance, blueberry juice supplementation increased the serum and hepatic TG contents, decreased the thermogenesis markers in iBAT, downregulated ER stress response and arrested autophagy in the liver of prediabetic rats, without effects on body weight gain and adiposity. Considering the pivotal role on the lipid management of the mechanisms assessed (thermogenesis, hepatic autophagy and ER stress response, as well as gut microbiota and SCFAs composition), and despite the incapability to mechanistically fully explain how BJ induced these alterations, we identify BJ-deleterious effects on these mechanisms as the main putative contributors to the worsening of the lipid profile. Nonetheless, considering the possibility that others unassessed factors could also be playing a role in lipid management, further studies should be performed in order to elucidate the precise cause(s) of the worsening in the lipid metabolism in the liver and adipose tissue, as well as the detailed impact on gut microbiota, in order to clarify whether these effects will affect the course of the disease when used at this stage of prediabetes.

## Figures and Tables

**Table 1:** Primer sequences and real-time PCR conditions used for gut microbiota analysis.

Target Group	Primer Sequence (5′-3′)	Genomic DNA Standard	PCR Product Size (bp)	AT (°C)	Ref.
*Firmicutes*	AGC TGA CGA CAA CCA TGC AC ATG TGG TTT AAT TCG AAG CA	*Lactobacillus gasseri* ATCC 33323	126	45	[21]
*Lactobacillus*	AGC AGT AGG GAA TCT TCC A CAC CGC TAC ACA TGG AG	*Lactobacillus gasseri* ATCC 33323	351	55	[22]
*Enterococcus*	ACT CGT TGT ACT TCC CTT GT CCC TTA TTG TTA GTT GCC ATC ATT	*Enterococcus gilvus* ATCC BAA-350	155	50	[23]
*Clostridium* *leptum*	CTT CCT CCG TTT TGT CAA GCA CAA GCA GTG GAG T	*Clostridium leptum* ATCC 29065	239	45	[23]
*Roseburia*	CGG CAC CGA AGA GCA AT TAC TGC ATT GGA AAC TGT CG	*Roseburia hominis* A2-183	230	50	[23]
*Bacteroidetes*	AGC TGA CGA CAA CCA TGC AG CAT GTG GTT TAA TTC GAT	*Bacteroides vulgatus* ATCC 8482	126	45	[21]
*Prevotella*	GGT CGG GTT GCA GAC C CAC RGT AAA CGA TGG ATG CC	*Prevotella nigrescens* ATCC 33563	513	50	[24]
*Bacteroides*	CCA GTA TCA ACT GCA ATT TTA ATA GCC TTT CGA AAG RAA GAT	*Bacteroides vulgatus* ATCC 8482	495	45	[24]
*Bifidobacterium*	CCA GTA TCA ACT GCA ATT TTA ATA GCC TTT CGA AAG RAA GAT	*Bifidobacterium* *longum subsp. Infantis* ATCC 15697	244	50	[21]

AT: annealing temperature; bp: base pairs. PCR: polymerase chain reaction.

**Table 2:** Primary and secondary antibodies used in Western blot analysis.

Antibody	Reference	Source	Animal Origin	Blocking Solution	Solution	Dilution	Incubation Time
Primary Antibody							
Anti-PGC1α	Ab191838	Abcam	Rabbit	5% Milk in TBS-T	1% Milk in TBS-T	1:1500	48 h
Anti-UCP1	Ab23841	Abcam	Rabbit	5% Milk in TBS-T	1% Milk in TBS-T	1:1500	24 h
Anti-eIF2α	5324	Cell Signaling Technology	Rabbit	5% BSA in TBS-T	1% BSA in TBS-T	1:2000	24 h
Anti-IRE1α	3294	Cell Signaling Technology	Rabbit	5% BSA in TBS-T	1% BSA in TBS-T	1:1000	24 h
Anti-CHOP	2895	Cell Signaling Technology	Mouse	5% BSA in TBS-T	1% BSA in TBS-T	1:1000	24 h
Anti-SQSTMI/p62	5114S	Cell Signaling Technology	Rabbit	5% BSA in TBS-T	1% BSA in TBS-T	1:1000	24 h
Anti-LC3	PA1-16931	Thermo Fisher Scientific	Rabbit	5% BSA in TBS-T	1% BSA in TBS-T	1:1000	24 h
Loading Controls							
Anti-β-actin	AB0145-200	Sicgen	Goat	5% BSA in TBS-T	1% BSA in TBS-T	1:1000	24 h
Anti-Calnexin	AB0041-200	Sicgen	Goat	5% BSA in TBS-T	1% BSA in TBS-T	1:1000	24 h
Anti-GAPDH	AB0049-200	Sicgen	Goat	5% BSA in TBS-T	1% BSA in TBS-T	1:5000	24 h
Secondary Antibodies							
Anti-Rabbit	R-0572-050	Advansta	Goat	Same as respective antibodies		1:10,000	1 h
Anti-Mouse	R-05071-500	Advansta	Goat			1:10,000	1 h
Anti-Goat	AB1011-1000	Sicgen	Goat			1:10,000	1 h

BSA: bovine serum albumin; CHOP: C/EBP homologous protein; eIF2α: eukaryotic initiation factor 2-α; GAPDH: glyceraldehyde 3-phosphate dehydrogenase; IRE1α: Inositol-requiring enzyme 1α; LC3: microtubule-associated protein 1 light chain 3; PGC1α: peroxisome proliferator-activated receptor-gamma coactivator-1alpha; SQSTMI/p62: sequestosome-1/protein p62; TBS-T: tris-buffered saline with Tween-20; UCP1: uncoupling protein-1.

**Table 3:** Body and tissue weights, food, beverage and caloric intake.

	CD	HFD	HFD + BJ
Body and Tissue Weights			
Δ BW (g)	121.600 ± 4.202	202.286 ± 23.550 *	204.250 ± 18.890 *
Δ BW (%)	32.600 ± 1.166	53.143 ± 6.212 *	50.857 ± 3.575 *
eWAT (g)	10.047 ± 0.544	23.936 ± 1.762 ***	22.928 ± 2.221 ***
eWAT/BW	2.044 ± 0.135	4.733 ± 0.374 ****	4.199 ± 0.289 ***
iBAT (g)	0.707 ± 0.011	0.925 ± 0.062	0.947 ± 0.075 *
iBAT/BW	0.146 ± 0.004	0.173 ± 0.014	0.175 ± 0.012
Liver (g)	15.780 ± 0.028	15.387 ± 0.853	16.359 ± 0.973
Liver/BW	3.384 ± 0.097	2.550 ± 0.138 ***	2.831 ± 0.080 **
Food and Beverage Intake			
Food (g/week)	145.833 ± 2.027	104.127 ± 4.400 ****	105.797 ± 4.162 ****
Beverage (mL/week)	198.750 ± 2.294	176.453 ± 5.435 **	228.047 ± 8.098 ^####^
Calories (Kcal/week)			
Carbohydrates	311.916 ± 4.334	199.185 ± 8.412 ****	210.557 ± 7.634 ****
Lipids	39.506 ± 0.551	218.272 ± 9.220 ****	221.773 ± 8.725 ****
Proteins	107.953 ± 1.500	71.942 ± 3.039 ****	73.095 ± 2.874 ****
Total	459.375 ± 6.385	489.399 ± 20.670	505.425 ± 19.020

BW: body weight; eWAT: epididymal white adipose tissue; iBAT: interscapular brown adipose tissue; data are expressed as mean ± SEM (n = 5–8 per group). * *p* < 0.05, ** *p* < 0.01, *** *p* < 0.001 and **** *p* < 0.0001 vs. CD; #### *p* < 0.0001 vs. HFD, using a one-way ANOVA followed by a Tukey multiple comparison test.

**Table 4:** Gut microbiota composition and short-chain fatty acids.

	CD	HFD	HFD + BJ
Gut Microbiota Composition (Log10 copies/ng of DNA)			
*Firmicutes*	4.882 ± 0.547	6.678 ± 0.247 **	4.550 ± 0.157 ^##^
*Bacteroidetes*	4.228 ± 0.252	4.146 ± 0.129	4.504 ± 0.511
*Firmicutes/Bacteroidetes*	1.276 ± 0.035	1.590 ± 0.062 *	1.078 ± 0.095 ^###^
*Bifidobacterium* spp.	3.230 ± 0.594	1.768 ± 0.130	1.452 ± 0.153*
*Lactobacillus* spp.	4.828 ± 0.245	3.828 ± 0.100 **	3.899 ± 0.183*
*Prevotella* spp.	3.203 ± 0.417	1.437 ± 0.119 **	2.139 ± 0.228
*Bacteroides* spp.	2.977 ± 0.062	2.902 ± 0.332	2.086 ± 0.230
*Clostridium Leptum*	3.165 ± 0.114	3.308 ± 0.260	2.680 ± 0.130
*Enterococcus* spp.	2.583 ± 0.177	2.813 ± 0.161	3.068 ± 0.233
*Roseburia*	2.234 ± 0.306	2.038 ± 0.175	1.764 ± 0.453
Short-Chain Fatty Acids (µmol/g)			
C2 Acetic	113.230 ± 23.730	56.566 ± 9.231 *	52.059 ± 6.625 *
C3 Propionic	10.225 ± 2.441	7.095 ± 1.631	3.449 ± 0.554 *
C4 Butyric	6.182 ± 1.323	4.129 ± 1.186	2.450 ± 0.478 *
iC4 Isobutyric	0.574 ± 0.050	0.291 ± 0.114	0.183 ± 0.033 *
iC5 Isovaleric	0.858 ± 0.088	0.246 ± 0.034 *	0.220 ± 0.018 **
C5 Valeric	0.820 ± 0.048	0.541 ± 0.124	0.308 ± 0.046 ***
C6 Caproic	0.929 ± 0.148	0.430 ± 0.062 **	0.379 ± 0.039 **

Data are expressed as mean ± SEM (n = 3–7 per group). * *p* < 0.05, ** *p* < 0.01 and *** *p* < 0.001 vs. CD; ## *p* < 0.01 and ### *p* < 0.001 vs. HFD, using a one-way ANOVA followed by a Tukey multiple comparison test.

**Table 5:** Serum lipid profile.

Serum (mg/dL)	CD	HFD	HFD + BJ
Triglycerides	237.000 ± 27.180	135.333 ± 17.920	242.500 ± 29.420 ^#^
LDL-c	4.000 ± 0.548	7.714 ± 0.865 *	7.625 ± 0.800 *
HDL-c	24.200 ± 1.828	27.857 ± 2.539	31.000 ± 1.604
Total cholesterol	74.800 ± 6.070	80.857 ± 5.869	95.500 ± 4.424 *

HDL-c: high-density lipoprotein cholesterol; LDL-c: low-density lipoprotein cholesterol; data are expressed as mean ± SEM (n = 5–8 per group). * *p* < 0.05 vs. CD; # *p* < 0.05 vs. HFD, using a one-way ANOVA followed by a Tukey multiple-comparison test.

## Data Availability

Data is unavailable due to privacy restrictions.

## Supplementary Materials

The following supporting information can be downloaded at: https://www.mdpi.com/article/10.3390/nu16040513/s1, Figure S1: Chromatographic profile of phenolic compounds in BJ, obtained with HPLC-PDA (320/530 nm); Figure S2: Chromatograms of PDA, UV (channel A, 280 nm and channel B, 320 nm) and MS (TIC). Table S1: Compounds identified in BJ by HPLC-PDA-ESI-MSn.
