# Supplementary material for: Effect of Blueberry Supplementation on a Diet-Induced Rat Model of Prediabetes—Focus on Hepatic Lipid Deposition, Endoplasmic Stress Response and Autophagy
Source: Nutrients. 2024 Feb 13;16(4):513. doi: 10.3390/nu16040513 (PMC10892331; doi:10.3390/nu16040513)
Supplement: Supplementary file 1 [file nutrients-16-00513-s001.zip › nutrients-2836854-supplementary.pdf]

Supplementary Data

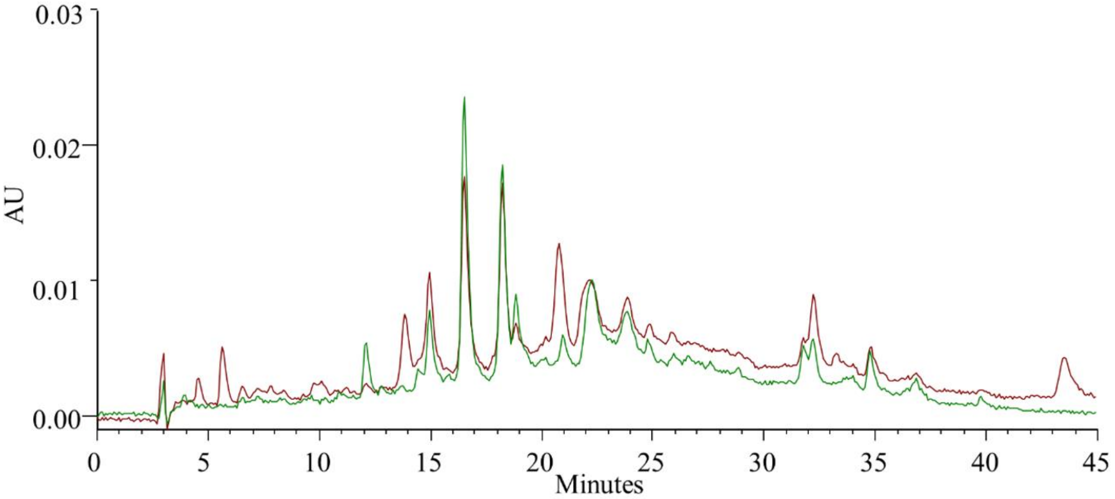

Figure S1: Chromatographic profile of phenolic compounds in BJ, obtained with HPLC-PDA (320 / 530 nm). Liquid chromatograph with a photodiode spectrophotometer-PDA detector (Thermo Finnigan Surveyor, San Diego, CA, USA) interfaced with a linear ion trap mass spectrometer (LIT-MS) (LTQ XL, Thermo Scientific, Waltham, MA, USA). The sample was injected on a Spherisorb ODS-2 column (150x2.1 mm id; particle size, 3 µm; Waters Corp., Milford, MA, USA) with a Spherisorb ODS-2 guard cartridge (10x4.6 mm id; particle size, 5 µm; Waters Corp., Milford, MA, USA) at 25 ºC.

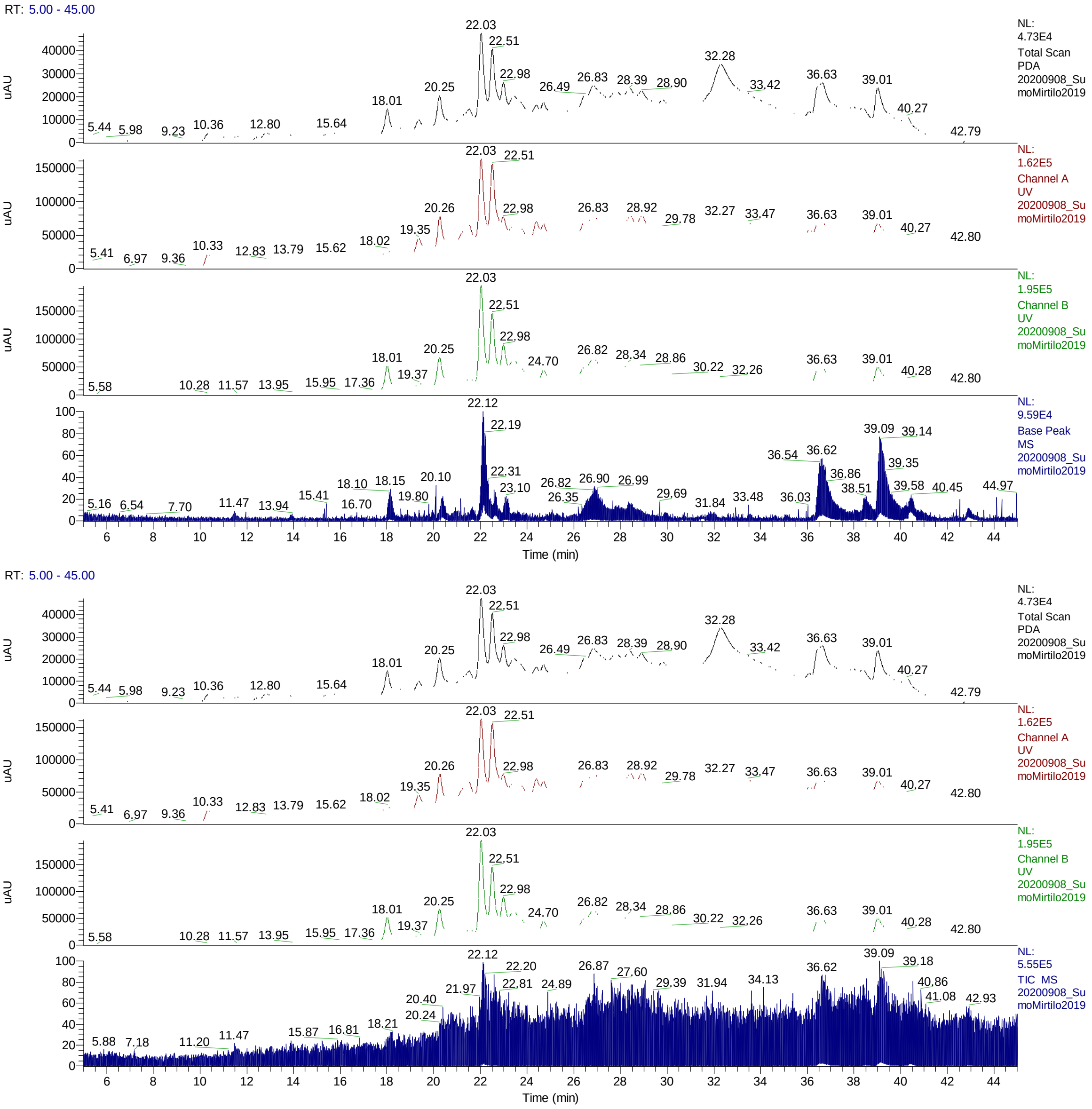

Figure S2: Chromatograms of PDA, UV (channel A, 280nm and channel B, 320nm) and MS (TIC). The elution was performed using 1% aqueous formic acid (v/v) (A) and methanol (B) as mobile phase, with a gradient profile of 0–75 min (0%–100% B), at a flow rate of 200 µLmin-1. The PDA detection was recorded in a wavelength range of 200–600 nm, followed by the detection in the mass spectrometer. Mass spectra were acquired in a negative ion mode. The mass spectrometer performed three consecutive scans: Full mass (m/z 100–2000), MS2 of the most abundant ion in the full mass, and MS3 of the most abundant ion in the MS2. Source and capillary voltage were 5.0 kV and -35.0 V, respectively. Capillary temperature was 275ºC. Nitrogen was used as sheath and auxiliary gas at 40 and 5 Finnigan arbitrary units, respectively, and helium as collision gas with a normalized energy of 35%. Data treatment was carried out with the XCALIBUR software (Thermo Scientific, Waltham, MA, USA).

Table S1: Compounds identified in BJ by HPLC-PDA-ESI-MS^n.

Peak	R_t (min)	$\lambda_{máx}$ (nm)	ESI-MSn[a] [*m/z* (relative abundance, %)]			Attempt to identify (reference)
			Precursor Ion $[M-H]^-$	MS^2	MS^3	
**1**	13.85	223, 261	**315** (100)	**153** (100)	**123** (100)	**Protocatechuic acid hexose** (Fang, Yu e Prior, 2002)
**2**	18.01	222, 251sh, 260, 329	**355** (100)	247 (11), 235 (11), 207 (16), **193** (100), 192 (32), 165 (10)	**165** (100)	**Ferulic acid hexose** (Fang, Yu e Prior, 2002)
**3**	20.25	222, 246, 286, 291sh, 299sh, 321sh	**341**	**179** (100)	**135** (100)	**Caffeic acid hexose** (Fang, Yu e Prior, 2002)
**4**	21.46	222, 249, 286, 299sh, 332sh	**341**	**179** (100)	**135** (100)	**Caffeic acid hexose** (Fang, Yu e Prior, 2002)
**5**	22.03	222, 240, 291sh, 301sh, 313	**341**	**179** (100)	**135** (100)	**Caffeic acid hexose** (Fang, Yu e Prior, 2002)
**6**	22.51	222, 243, 291, 229sh, 317sh	**401**	**355** (100), 193 (16)	**193** (100)	**Ferulic acid hexose** (Fang, Yu e Prior, 2002)
**7**	22.98	220, 246, 291sh, 299sh, 323	**355**	217 (48), **193** (100), 175 (20)	178 (27), 149 (54), **134** (100)	**Ferulic acid hexose** (Fang, Yu e Prior, 2002)
**8**	23.44	222, 247, 291, 299sh, 318	**353**	295 (20), **191** (100)	173 (53), 171 (23), 155 (17), **127** (100), 111 (33), 109 (27), 93 (24), 87 (15), 85 (47)	**5-CQA** (Clifford, Knight e Kuhnert, 2005)
**9**	24.39	222, 249sh, 273, 298sh, 330sh	**431**	**385** (100), 223 (10)	**223** (100)	**Sinap acid hexoside** (Bujor *et al.*, 2016)
**10**	26.3	221, 248, 273, 299sh, 330sh	**861**	843 (11), 699 (70), 681 (21), 669 (56), 533 (25), 509 (81), 507 (32), **353** (100), 351 (73), 345 (14)	**191** (100)	**Caffeoyl-hidroxydihidro-CQA derivative** (Matei, Jaiswal e Kuhnert, 2012)
**11**	26.56	221, 246, 280, 299sh, 327sh	**861**	699 (35), 669 (40), 533 (45), 509 (56), 507 (26), **353** (100), 351 (42)	**191** (100)	**Caffeoyl-hidroxydihidro-CQA derivative** (Matei, Jaiswal e Kuhnert, 2012)
**12**	26.83	222, 245, 283,	**861**	699 (48), 695 (11), 669 (52), 533 (46),	**191** (100)	**Caffeoyl-hidroxydihidro-CQA derivative**

Peak	R_t (min)	$λ_{máx}$ (nm)	ESI-MSn[a] [*m/z* (relative abundance, %)]			Attempt to identify (reference)
			Precursor Ion [M-H]-	MS²	MS³	
		299sh, 325sh		509 (72), 507 (38), **353** (100), 351 (78)		(Matei, Jaiswal e Kuhnert, 2012)
**13**	27.77	222, 246, 280, 299sh, 323sh	**861**	843 (47), 699 (35), 695 (10), 681 (17), 669 (68), 651 (58), 583 (19), 533 (28), 509 (59), 507 (41), 489 (17), **353** (100), 351 (52), 345 (10)	**191** (100)	**Caffeoyl-hidroxydihidro-CQA derivative** (Matei, Jaiswal e Kuhnert, 2012)
**14**	28.39	221, 246, 274, 299sh, 328sh	**861**	829 (23), 699 (40), 681 (15), 669 (41), 533 (41), 515 (13), 509 (44), 507 (23), **353** (100), 351 (40), 345 (14)	**191** (100)	**Caffeoyl-hidroxydihidro-CQA derivative** (Matei, Jaiswal e Kuhnert, 2012)
**15**	28.90	221, 247, 276, 299sh, 327sh	**831**	699 (46), 639 (35), 533 (35), 507 (25), 489 (10), 479 (45), **353** (100), 351 (57), 345 (14)	**191** (100)	**Caffeoyl-hidroxydihidro-CQA derivative** (Matei, Jaiswal e Kuhnert, 2012)
**16**	32.28	222, 246, 283	**509**	**463** (100)	**331** (100), 161 (22)	**Laricitrin–O–pentoside** (Lätti *et al.*, 2010)
**17**	36.63	220, 252, 267sh, 287sh, 349	**463**	**301** (100)	273 (13), 257 (14), **179** (100), 151 (59)	**Quercetin-O–hexoside** (Lätti *et al.*, 2010)
**18**	37.95	251, 270sh, 336, 530	**433**	**301** (100)	273 (19), 272 (18), 257 (15), 256 (10), **179** (100), 151 (71)	**Quercetin-O-pentoside** (Lätti *et al.*, 2010)
**19**	37.95	251, 270sh, 336, 530	---	---	---	**Malvidin derivative** (Howard *et al.*, 2016)
**20**	38.36	252, 268sh, 343, 531sh	**433**	**301** (100)	273 (15), **179** (100), 151 (74)	**Quercetin -O-pentoside** (Lätti *et al.*, 2010)
**21**	38.36	252, 268sh, 343, 531sh	---	---	---	**Delphinidin derivative** (Howard *et al.*, 2016)
**22**	39.01	220, 254, 266sh, 299sh, 347	**447**	**301** (100)	283 (12), 273 (19), **179** (100), 151 (71)	**Quercetin - O-deoxyhexoside** (Lätti *et al.*, 2010)
**23**	40.27	253, 269sh, 299sh, 349	**623**	**315** (100), 300 (19)	**300** (100)	**Isorhamnetin - hexose-deoxyhexoside** (Simirgiotis e Schmeda-Hirschmann, 2010)

**Peak**	**R_t** (min)	**$λ_{máx}$** (nm)	**ESI-MSn[a]** [*m/z* (relative abundance, %)]			**Attempt to identify** (reference)
			**Precursor Ion $[M-H]^-$**	**MS^2**	**MS^3**	
**24**	42.79	252, 268sh, 343, 531sh	---	---	---	**Malvidin** (Howard *et al.*, 2016)

Identification based on the UV-Vis spectra, the molecular weight and the fragmentation patterns, which are according to authors. The base peaks in MS spectra are in bold.

Bibliography

1. BUJOR, Oana Crina *et al.* - Seasonal variations of the phenolic constituents in bilberry (Vaccinium myrtillus L.) leaves, stems and fruits, and their antioxidant activity. **Food Chemistry**. . ISSN 18737072. 213:2016) 58–68. doi: 10.1016/j.foodchem.2016.06.042.
2. CLIFFORD, Michael N. *et al.* - Hierarchical Scheme for LC-MS n Identification of Chlorogenic Acids. 2003). doi: 10.1021/jf026187q.
3. CLIFFORD, Michael N.; KNIGHT, Susan; KUHNERT, Nikolai - Discriminating between the six isomers of dicaffeoylquinic acid by LC-MSn. **Journal of Agricultural and Food Chemistry**. . ISSN 00218561. 53:10 (2005) 3821–3832. doi: 10.1021/jf050046h.
4. CLIFFORD, Michael N.; KNIGHT, Susan; KUHNERT, Nikolai - Discriminating between the Six Isomers of Dicaffeoylquinic Acid by LC-MS n. 2005). doi: 10.1021/jf050046h.
5. FANG, Nianbai; YU, Shanggong; PRIOR, Ronald L. - LC/MS/MS characterization of phenolic constituents in dried plums. **Journal of Agricultural and Food Chemistry**. . ISSN 00218561. 50:12 (2002) 3579–3585. doi: 10.1021/jf0201327.
6. HOWARD, Luke R. *et al.* - Improved stability of blueberry juice anthocyanins by acidification and refrigeration. **Journal of Berry Research**. . ISSN 18785123. 6:2 (2016) 189–201. doi: 10.3233/JBR-160133.
7. LÄTTI, Anja K. *et al.* - Anthocyanin and flavonol variation in bog bilberries (Vaccinium uliginosum L.) in Finland. **Journal of Agricultural and Food Chemistry**. . ISSN 00218561. 58:1 (2010) 427–433. doi: 10.1021/jf903033m.
8. MATEI, Marius Febi; JAISWAL, Rakesh; KUHNERT, Nikolai - Investigating the chemical changes of chlorogenic acids during coffee brewing: Conjugate addition of water to the olefinic moiety of chlorogenic acids and their quinides. **Journal of Agricultural and Food Chemistry**. . ISSN 00218561. 60:49 (2012) 12105–12115. doi: 10.1021/jf3028599.
9. SIMIRGIOTIS, M. J.; SCHMEDA-HIRSCHMANN, G. - Direct identification of phenolic constituents in Boldo Folium (Peumus boldus Mol.) infusions by high-performance liquid chromatography with diode array detection and electrospray ionization tandem mass spectrometry. **Journal of Chromatography A**. . ISSN 00219673. 1217:4 (2010) 443–449. doi: 10.1016/j.chroma.2009.11.014.
